# Mating Stimulates the Immune Response and Sperm Storage-Related Genes Expression in Spermathecae of Bumblebee (*Bombus terrestris*) Queen

**DOI:** 10.3389/fgene.2021.795669

**Published:** 2021-11-26

**Authors:** Yueqin Guo, Qi Zhang, Xiao Hu, Chunxiu Pang, Jilian Li, Jiaxing Huang

**Affiliations:** ^1^ Key Laboratory for Insect-Pollinator Biology of the Ministry of Agriculture and Rural Affairs, Institute of Apicultural Research, Chinese Academy of Agricultural Sciences, Beijing, China; ^2^ College of Animal Science, Shanxi Agricultural University, Taigu, China; ^3^ College of Animal Science and Technology, Yunnan Agricultural University, Kunming, China

**Keywords:** bumblebee, *Bombus terrestris*, spermathecae, transcriptome, RT-qPCR, gene expression

## Abstract

Bumblebee queens have remarkable spermathecae that store sperm for year-round reproduction. The spermathecal gland is regarded as a secretory organ that could benefit sperm storage. Queen mating provokes substantial physiological, behavioral, and gene expression changes. Here, the transcriptomes of spermathecae were compared between virgins and mated queens of the bumblebee, *Bombus terrestris* L., at 24 h post mating. Differentially expressed genes were further validated by real time quantitative PCR and immunofluorescence assay. In total, the expression of 11, 069 and 10, 862 genes were identified in virgins and mated queens, respectively. We identified that 176 differentially expressed genes between virgin and mated queen spermathecae: 110 (62.5%) genes were upregulated, and 66 (37.5%) genes were downregulated in mated queens. Most of the differentially expressed genes validated by RT-qPCR were concentrated on immune response [i.e., leucine-rich repeat-containing protein 70 (35.8-fold), phenoloxidase 2 (41.9-fold), and defensin (4.9-fold)] and sperm storage [i.e., chymotrypsin inhibitor (6.2-fold), trehalose transporter Tret1 (1.7-, 1.9-, 2.4-, and 2.4-fold), and heterogeneous nuclear ribonucleoprotein A3 (1.2-, and 2.6-fold)] functions in the spermathecae of mated queens. Procollagen-lysine, 2-oxoglutarate 5-dioxygenase 1 (PLOD1) was hypothesized to promote the mating behavior according to RT-qPCR and immunofluorescence assay. The expression levels of most upregulated immune genes were decreased significantly at 3 days post mating. In conclusion, the external sperm transfer into spermathecae led to the significantly upregulated immune response genes in bumblebees. These gene expression differences in queen spermathecae contribute to understanding the bumblebee post mating regulatory network.

## Introduction

Long-term sperm storage by reproductive females is common in eusocial insects, such as bumblebees, honeybees, and ants, whereby queens typically mate early in life, store sperm in their spermatheca, and subsequently use the stored sperm throughout their lifetimes (Schoeters and Billen 2000; Chérasse and Aron 2018; Rangel et al., 2021). Insect spermathecae have associated secretory cells (spermathecal secretory cells, SSCs) that produce nutrients involved in sperm storage (Wolfner 2011; Sun and Spradling 2013; Pascini and Martins 2017; Pascini et al., 2020). The spermathecal gland is regarded as a secretory organ and might also function as an additional sperm storage organ. In the spermathecae, there is a more complete protein network that is, conducive to long-term sperm storage (Baer et al., 2009a; Baer et al., 2009b). Hundreds of proteins representing the main components of spermathecal fluid have been identified. They belong to a series of different functional groups, the most obvious of which are energy metabolism enzymes and antioxidant defense enzymes. The male seminal fluid has proven to be essential for long-term sperm storage (Jasper et al., 2020). Therefore, mating leads to the biochemical and physiological changes the spermathecae of insects (Baer et al., 2009a; Huo et al., 2020).

Mating is fundamental to the success and reproduction in organisms and has effects on female biology and behavior (Gomulski et al., 2012; Short and Lazzaro 2013; Alfonso-Parra et al., 2016). Mating not only allows females to obtain sperm but also delivers some seminal fluid proteins are delivered to females (Huo et al., 2020). After mating in *Cremato-gaster* (Gotoh et al., 2017), and *Anopheles* (Shaw et al., 2014), the ant *Atta colombica* (Dosselli et al., 2019), *Drosophila* (Prokupek et al., 2009), *Apis mellifera* (Baer et al., 2009a; Liu et al., 2020), molecules involved in the carbohydrate and lipid metabolism, cellular transport, immune response, and oxidative stress have been identified in the sperm storage organs and might play protective roles in sperm and/or mediate female post-mating processes. Female reproductive fluid (FRF), has exerted positive phenotypic effects on sperm competition in males, including chemoattraction, and alterations in sperm velocity (Gasparini et al., 2020). The spermathecae of honeybee (*A. mellifera*) possesses some important proteins, such as glutathione-transferase, catalase, thioredoxin 2, and thioredoxin reductase 1, kielin/chordin-like and trehalase, that can significantly improve sperm motility and are involved in the long-term maintenance of stored sperm (den Boer et al., 2009; Gotoh et al., 2017; Rangel et al., 2021). The components in the seminal fluid of honeybee (*A. mellifera*), such as seminal fluid proteins (SFPs), are largely responsible for stimulating post-mating changes in queens (Jasper et al., 2020).

Up to now, no reports have focused on the molecular mechanisms maintaining the sperm viability for years inside bumblebee queens spermathecae, although sperm storage plays important functions in bumblebee reproduction. Here, the gene expression in the spermathecae of bumblebee queens post mating was characterized by RNA-sequencing. The differentially expressed genes (DEGs) were further validated by real time quantitative PCR (RT-qPCR) and immunofluorescence (IF) assay. Most of the DEGs, such as leucine-rich repeat-containing protein 70 (LRRC70, LRR), phenoloxidase 2 (PO2), defensin (Def), including chymotrypsin inhibitor (trypsin inhibitor, TIL), trehalose transporter (Tret1), and heterogeneous nuclear ribonucleoprotein A3 (hnRNP) were concentrated on immune response and sperm storage function. Among these genes, a slight decrease in procollagen-lysine, 2-oxoglutarate 5-dioxygenase 1 (PLOD1) was observed in the mated queen spermathecae with RT-qPCR and IF. These gene expression differences in queen spermathecae caused by mating contribute to understand the regulatory network of bumblebees post mating.

## Materials and Methods

### Samples Collection

**FIGURE 1:**
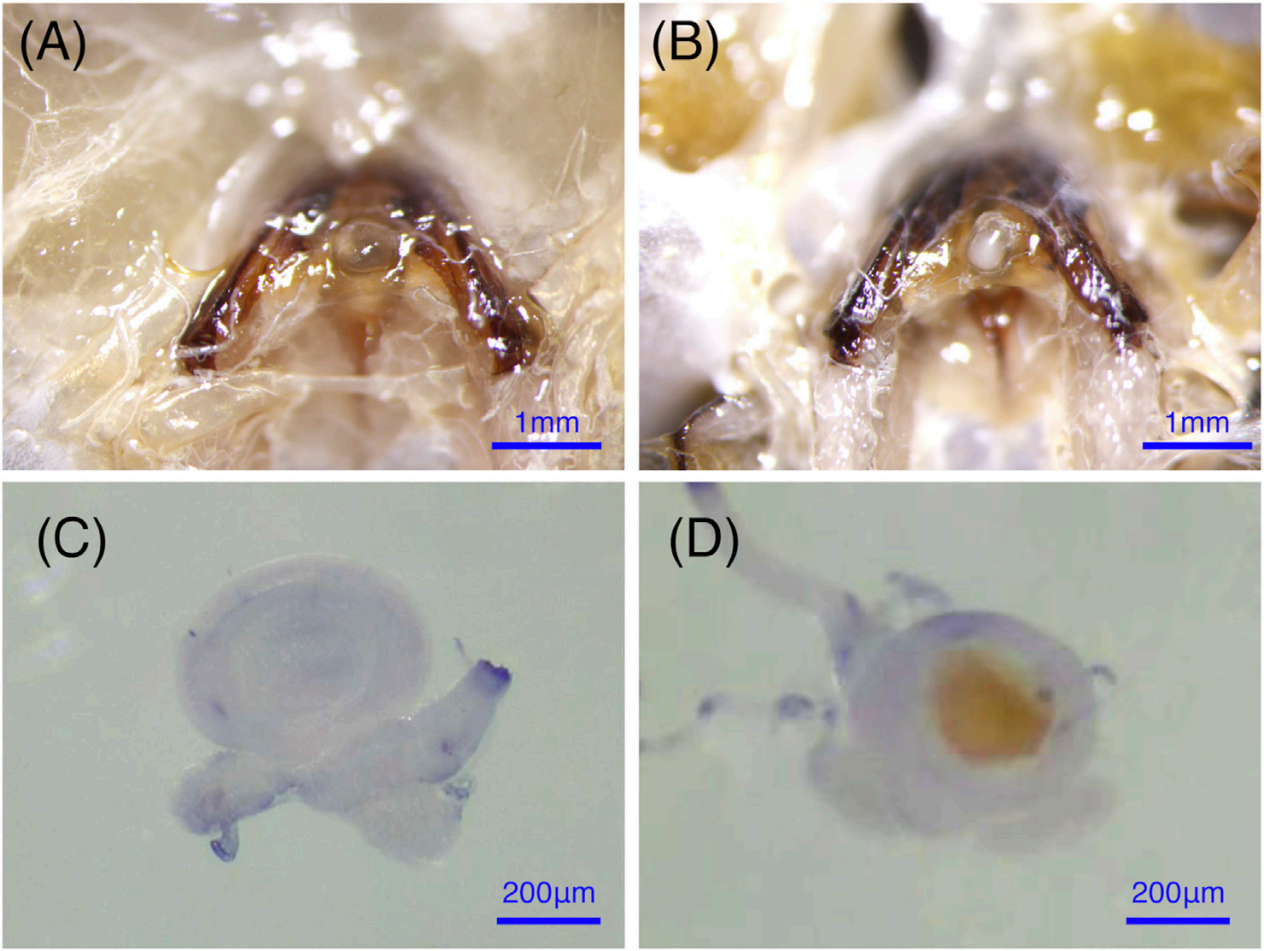
The anatomical structure of the spermathecae in virgin and mated bumblebee queens. **(A)** and **(C)** show that the spermathecae of the virgin queen are empty and almost translucent. **(B)** and **(D)** show that the mated queens spermathecae is filled with semen.

Bumblebees (*B. terrestris*) were collected from the rearing room at the Institute of Apicultural Research, Chinese Academy of Agricultural Science, Beijing, China. Bumblebees were sampled from six independent colonies that were raised in an artificial breeding room (in constant darkness with a temperature of 28 ± 0.5°C and 60 ± 5% relative humidity) and fed fresh frozen pollen and a 50% sugar solution every other day (Gurel and Gosterit 2008). The new emerged queens were aged for 7 days, and half of them were mated with the males, to ensure different stages of virgin and mated queens were collected at the same days old. The queens were kept at room temperature until they were dissected for the collection of the spermathecae. The spermathecae is empty and almost translucent if the bumblebee queen is unfertilized. After mating, the sperm in the spermathecae form an obvious opaque white sphere, which is easy to see (Figure 1) (Duvoisin et al., 1999). The spermathecae of virgin and mating bumblebee queens to be used for morphological observation were dissected in 1 × PBS (10 mM NaH_2_PO4/Na_2_HPO4, 175 mM NaCl, pH 7.4, Solarbio, Beijing, China), and fixed in 4% paraformaldehyde for 25 min at room temperature, washed with 0.1% Triton X-100 in 1 X PBS (1 X PBT) for 2 × 5 min, and then mounted in mounting medium (90% glycerol). The images were performed with a Leica EZ4W microscope. The spermathecae to be used for RT-qPCR were dissected from the abdomen, and immediately frozen in liquid nitrogen. Tissues from ten bumblebees were pooled as a biological replicate. Three biological replicates were performed for each mating status. All samples were stored at −80°C until they were used for RNA extraction.

### RNA Extraction and cDNA Synthesis

Total RNA was extracted from the spermathecae of virgin and the mated queens with TRIzol reagent (Invitrogen, Carlsbad, CA, United States) following the manufacturer’s instructions. The purity of the RNA was assessed using a NanoDrop 2000 spectrophotometer (Thermo Fisher Scientific, Waltham, MA, United States) at 260/280 nm, and RNA integrity was screened by 1.5% (w/v) agarose gel electrophoresis. The first strand of cDNA was synthesized according to the instructions of the Reverse Transcription kit (Takara, Dalian, China). The reaction conditions were as follows: 42°C for 30 min, 99°C for 5 min, and 5°C for 5 min, and then the products were stored at −20°C until use.

### cDNA Library Construction, and Illumina Sequencing

RNA-sequencing was performed with three biological replicates consisting of three pooled spermatheca samples (*n* = 10 virgin or mated queens). Following the manufacturer’s recommendations, RNA-sequencing libraries were generated with the NEBNext Ultra™RNA Library Prep Kit from Illumina (NEB, Ipswich, MA, United States), and index codes were added to attribute the sequences to each sample. Using poly-T oligo-attached magnetic beads, mRNA was purified from total RNA. Fragmentation was carried out using divalent cations under high temperature in NEBNext First-Strand Synthesis Reaction Buffer (5 ×). First- and second-strand cDNA was successfully synthesized. The remaining overhangs were converted into blunt ends *via* exonuclease/polymerase treatment. The short fragments and adapters were linked together. The suitable fragments were chosen as templates, and the subsequent PCR amplification was performed with Phusion High-Fidelity DNA polymerase, universal PCR primers and an Index (X) Primer to obtain Index-coded samples. Finally, the PCR products were purified, and the quality of library was assessed on an Agilent Bioanalyzer 2100 system. Index-coded samples were prepared on a cBot Cluster Generation System using the TruSeq PE Cluster Kit v4-cBot-HS (Illumina). The library preparations were sequenced on the Illumina NovaSeq platform.

### RNA-Seq Data Analysis

Adaptors were removed from the raw reads, and low-quality reads were filtered out to obtain the clean reads with NGS QC Toolkit (version 2.3) and Cutadapt (version 1.16). After the quality control, the clean reads were mapped to the reference genome of *B. terrestris* (version 1.1.1) through Hisat2 software. Each sample was quantified with StringTie. The R package ballgown was used to acquire the gene expression levels. The gene expression level of each transcript was estimated with the fragments per kilobase of transcript per million mapped reads (FPKM) method. FPKM values were directly used to compare the differences of gene expression between various samples. The transcripts with a *p*-value ≤ 0.05 and the absolute value of the |log2 fold change| ≥ 1 were considered as differentially expressed genes (DEGs).

To annotate the DEGs, Blast2GO software was used to search against the nonredundant (NR) database in NCBI, Clusters of Orthologous Groups of Proteins (COG), Clusters of Orthologous Groups for Eukaryotic Complete Genomes (KOG), and Evolutionary Genealogy of Genes: Non-supervised Orthologous Groups (eggNOG) databases. Furthermore, Gene Ontology (GO terms) and Kyoto Encyclopedia of Genes and Genomes (KEGG pathway) analyses were performed with the default parameters.

### Quantitative PCR for Quantification of Candidate DEGs

Fifteen DEGs were selected as candidates to analyze their expression differences in the spermathecae of queens that response to mating by RT-qPCR using a Stratagene Mx3000 real-time PCR system (Agilent, United States). The primers were designed with Primer-BLAST (https://www.ncbi.nlm.nih.gov/tools/primer-blast/index.cgi), and the primer sequences are shown in Supplementary Table S1. First-strand cDNA samples were diluted (1:10 v/v) with DEPC-treated water. Amplification was carried out in a 20 µL reaction volume containing 10 µL of 10 × TB Green Master Mix (Takara, Dalian, China), 2 µL cDNA, and 0.5 µL of each primer at 10 µm. Quantitative measurements were normalized using β-actin and RP49. The qPCR conditions were as follows: 95°C for 30 s, followed by 40 cycles of 95°C for 5 s, and 63°C for 1 min. RT-qPCR was performed in duplicate on each of three independent biological replicates. All results are presented as the mean ± SEM of the biological replicates. The relative quantities of transcripts were calculated using the comparative Ct method (Livak and Schmittgen, 2001).

### Immunostaining and Fluorescence Microscopy

Immunostaining was performed as previously described (Li et al., 2013). Briefly, the spermathecae of mating and virgin bumblebee queens were dissected in 1 × PBS and fixed in 4% paraformaldehyde for 25 min at room temperature. Samples were rinsed, washed with 1 × PBT for 2 × 5 min and blocked in 3% BSA in 1 × PBT for 20 min. The primary antibody rabbit anti-PLOD antibody (ProteinTech Group, 1:400) was detected with a fluorescent-conjugated secondary antibody. Incubation with the secondary antibody was performed for 2 h at room temperature, and DAPI (Sigma; 0.1 mg/ml) and phalloidin (Cell Signaling Technology, CST; 8878S) were added during secondary antibody staining. The samples were mounted in mounting medium [70% glycerol containing 2.5% 1, 4-diazabicyclo (2.2.2) octane]. Confocal fluorescence imaging was performed with a Leica SP8 laser-scanning microscope (Leica). For quantification of PLOD1, fluorescence intensity was measured in ImageJ software (Figures 5, 6A).

### Phenoloxidase Activity Measurement

Spermathecae were homogenized in 1 × PBS. The supernatant was recovered by centrifugation at 3,000 g and 4°C for 10 min. The enzymatic activities of phenoloxidases (POs) in the spermathecae of bumblebee virgins or mated queens were determined with an insect PO ELISA kit (Abmart, AB-3369B, Shanghai, China), and the optical density of each well at 450 nm was measured with an ELISA reader (Molecular Devices SpectraMax i3, United States). The PO activities were calculated according to the calibration curve generated according to the manufacturer’s instructions. Three independent biological replicates were performed for each treatment.

### Statistical Analysis

Statistical analysis was performed using the Mann-Whitney *U* test or one-way ANOVA, and data are presented as the mean ± SEM. The graphs were created with R project software (version 4.0.5). The values and error bars presented in figures represent the means and standard errors of biological replicates.

## Results

### Sequencing Data and DEGs Analysis

**FIGURE 2:**
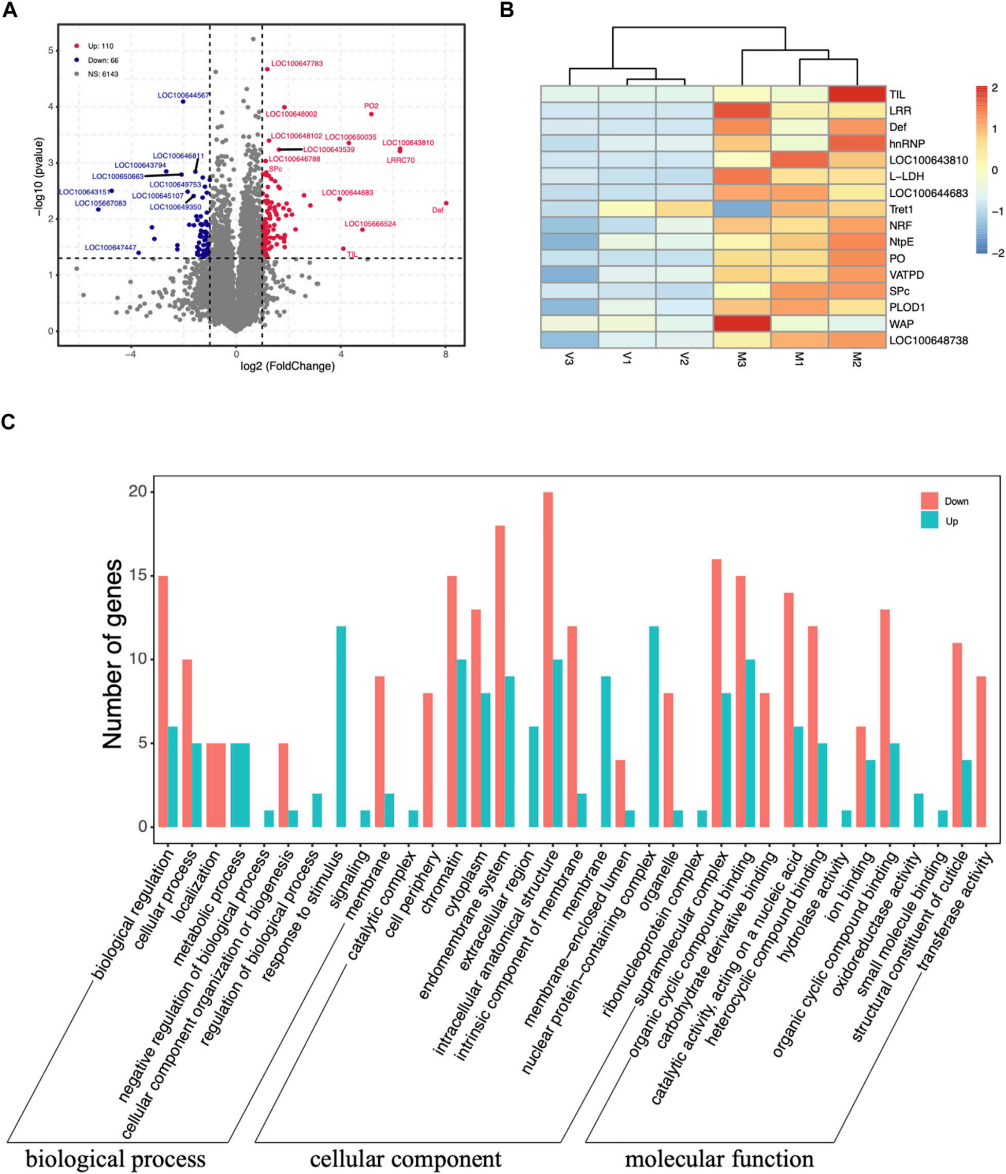
**(A)** Volcano plots displaying the upregulated (red dots) and downregulated (blue dots) differentially expressed genes between the spermathecae of mated and virgin bumblebee queens. Each dot represents one gene. The gray dots represent genes that were not differentially expressed [*p* > 0.05 and |log2 (fold-change)| ≤ 1]. **(B)** Expression profile of transcripts that were differentially expressed according to RNA-seq between mated and virgin bumblebee females at 24 h post mating. The heatmap shows the transcriptome data of the selected genes, which are based on the log2 (FPKM) values of genes in virgin and mated females. The color scale represents the scale of the log2 (FPKM) values. The tissues used in the analysis (*n* = 3 biological replicates per tissue type) were spermathecae from mated queens (“M”), and spermathecae from virgin queens (“V”). The color index at the top indicates the genes that were expressed at relatively low levels (blue) or at high levels (red) in each row. Heat maps were generated using the heatmap R package. **(C)** Distribution of up- and downregulated DEGs among the Gene Ontology (GO) terms in the biological process, cellular component, and molecular function categories.

The raw data quality statistics showed that the Q30 percentages value of the six samples was ranged from 89.07 to 93.65%. The percentages of rRNA were between 0.04 and 0.30% with an average 0.21%. The number of raw reads ranged from 23, 000, 077 to 31, 124, 050 with an average 25, 998, 530 reads. After the mapping of reads to the genome, 11, 069 genes in virgin and 10, 862 genes in mated queens were identified. The number of differentially expressed genes between virgin and mated queen spermathecae was 176. Among the DEGs, 110 (62.5%) were upregulated and 66 (37.5%) were downregulated (Figure 2A and Table 1).

**TABLE 1:** Statistics of the differentially expressed genes (DEGs).

Database	Total	BLAST	COG	eggNOG	KOG	GO	KEGG
DEGs	176	—	—	—	—	—	—
DEGs-annotated	108	146	61	147	112	80	31
Downregulation	66	45	20	54	40	31	19
Upregulation	110	101	41	93	72	49	12

**FIGURE 3:**
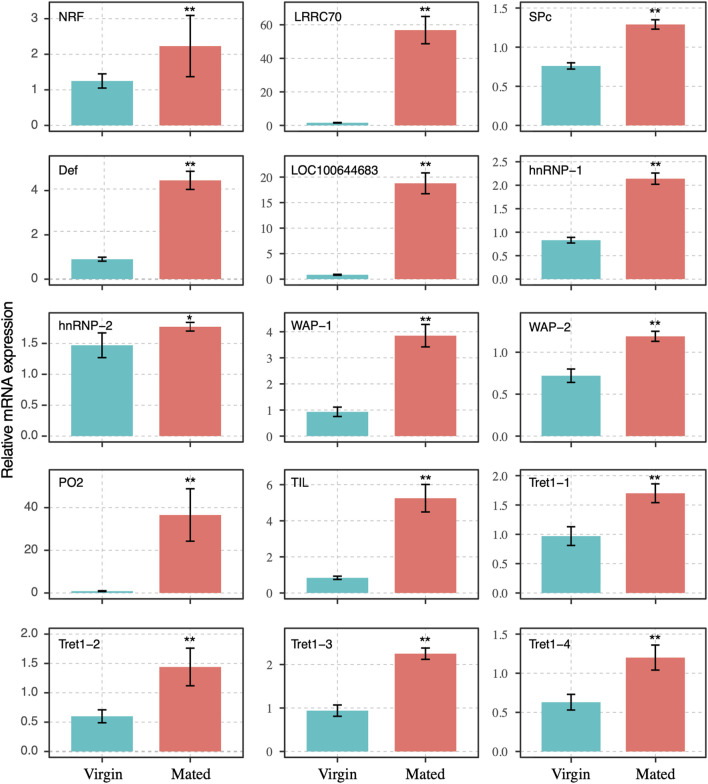
Expression profile of transcripts that were differentially expressed between mated and virgin bumblebee queens determined by RT-qPCR, at 24 h post mating. The expression levels (Mean ± SEM) of selected genes in virgin queens (blue bars) and mated queens (red bars). Asterisks indicate significant differences between the expression profiles of mated and virgin queens. *p < 0.05; **p < 0.01.

The clustering analysis based on the scale of DEGs FPKM values showed a satisfactory biological replication in the mated and virgin groups (Figure 2B). These genes, such as TIL, LRR, Def, hnRNP, LOC100643810, l-lactate dehydrogenase (L-LDH), LOC100644683, Tret1, nose resistant to fluoxetine protein 6 (NRF), V-type proton ATPase subunit E (NtpE), PO, V-type proton ATPase subunit D (VATPD), serine protease easter-like (SPc), PLOD1, waprin-Phi1-like (WAP), LOC100648738, were dramatically upregulated at 24 h post mating. Two novel genes, LOC100643810 (ncRNA) and LOC100644683 (mRNA), unique to the bumblebee *B. terrestris*, also showed significantly increased expression at 24 h post mating (Figures 2B, 3).

### Functional Annotation and Classification

The GO terms analysis showed that among the 110 upregulated DEGs, those related to nuclear protein-containing complex (12), chromatin (10), and intracellular anatomical structure (10) in cellular component terms were the most abundant, followed by those related to the response to stimulus (12) in biological process term, and the organic cyclic compound binding (10) and catalytic activity (6) in molecular function terms, (Figure 2C). In turn, the 66 downregulated DEGs were associated with the intracellular anatomical structure (20), endomembrane system (18), and supramolecular complex (16) terms (Figure 2C).

The DEGs were also mapped to canonical KEGG pathways to identify possible active biological pathways. Most genes in those pathways were upregulated. Specifically, the gluconeogenesis and neutrophil degranulation categories contained 13 upregulated DEGs, and the thiamine metabolism and purine metabolism were the next most enriched categories. The proton buffering model category contained more than 11 upregulated DEGs, molybdenum cofactor biosynthesis contained 9 upregulated DEGs, and hepatocellular carcinoma and pathways in cancer contained 5 upregulated DEGs, respectively (Table 2).

**TABLE 2:** Top 8 KEGG pathways of the differentially expressed genes (DEGs).

KEGG pathway	koID	Number of DEGs
Gluconeogenesis	R-DME-70263	13
Neutrophil degranulation	R-DME-6798695	13
Thiamine metabolism	ko00730	12
Purine metabolism	ko00230	11
The proton buffering model	R-RNO-167827	11
Molybdenum cofactor biosynthesis	R-HSA-947581	9
Hepatocellular carcinoma	ko05225	5
Pathways in cancer	ko05200	5

### Candidate DEGs RT-qPCR Validation

The transcript levels of 15 DEGs were selected for RT-qPCR analyses to confirm the validity of mating-related genes in the RNA-seq results, which revealed the upregulation of genes in one of the examined tissues (spermathecae of mated and virgin queens). The amplification efficiencies used for correction in all normalized fold-expression analyses ranged from 95.22 to 105.67%, which was within the acceptable range 90–110% (Bustin et al., 2009).

**FIGURE 4:**
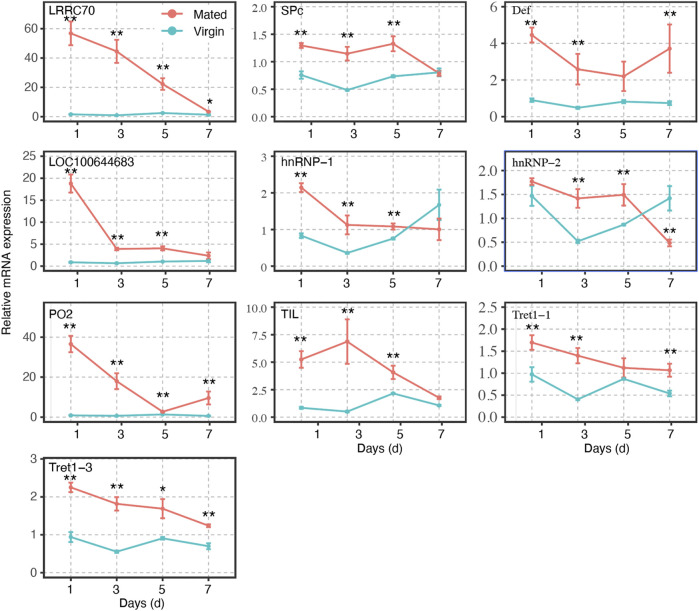
Gene expression profiles of selected mating-related DEGs at different time points in mated and virgin bumblebee queens at 1-, 3-, 5-, and 7- days, as determined by RT-qPCR. The expression levels (Mean ± SEM) of selected genes in virgin queens (blue lines) and mated queens (red lines). Asterisks indicate significant differences between the expression profiles of mated and virgin queens. *p < 0.05; **p < 0.01.

The transcript levels of gene NRF-6, LRRC70, SPc, Def, LOC100644683, hnRNP-1, hnRNP-2, WAP-1, WAP-2, PO2, TIL, and four transcripts of facilitated trehalose transporter genes (Tret1-1, Tret1-2, Tret1-3, and Tret1-4) were significant increased post mating at 24 h (Figure 3). These genes were chosen for RT-qPCR validation. The results of RNA-seq and RT-qPCR showed that 93.3% of these selected genes had consistent expression (Figures 2–4), with the exception of the PLOD1 gene. The correlation analysis showed that RT-qPCR and RNA-seq data was significantly correlated. The RT-qPCR data confirmed some of the differences in mRNA levels first identified in the RNA-sequencing data.

The transcripts of these genes in spermathecae were compared between virgins and mated queens of bumblebee, *B. terrestris* L., at 1-, 3-, 5-, and 7-days post mating. A total of ten mating-related DEGs (verified in RT-qPCR results at 24 h post mating) were used to verify the expression profiles at different time points within 7 days. The expression levels of the remaining genes gradually decreased. Interestingly, within 7 days, the expression levels of LRRC70, LOC100644683, and PO2, in mating females were gradually decreased post mating, and reached levels similar to those of virgin females, especially at 7 days (Figure 4). However, SPc, Def, Tret1-1, and Tret1-3 were always upregulated in mated females in comparison with virgin females (Figure 4). Although the expression level of TIL, hnRNP-1, and hnRNP-2 tended to fluctuate, they were still always higher than those of virgin females at 1, 3, and 5 days (Figure 4).

### PLOD1 Expression in the Spermathecae of Bumblebee Queens

**FIGURE 5:**
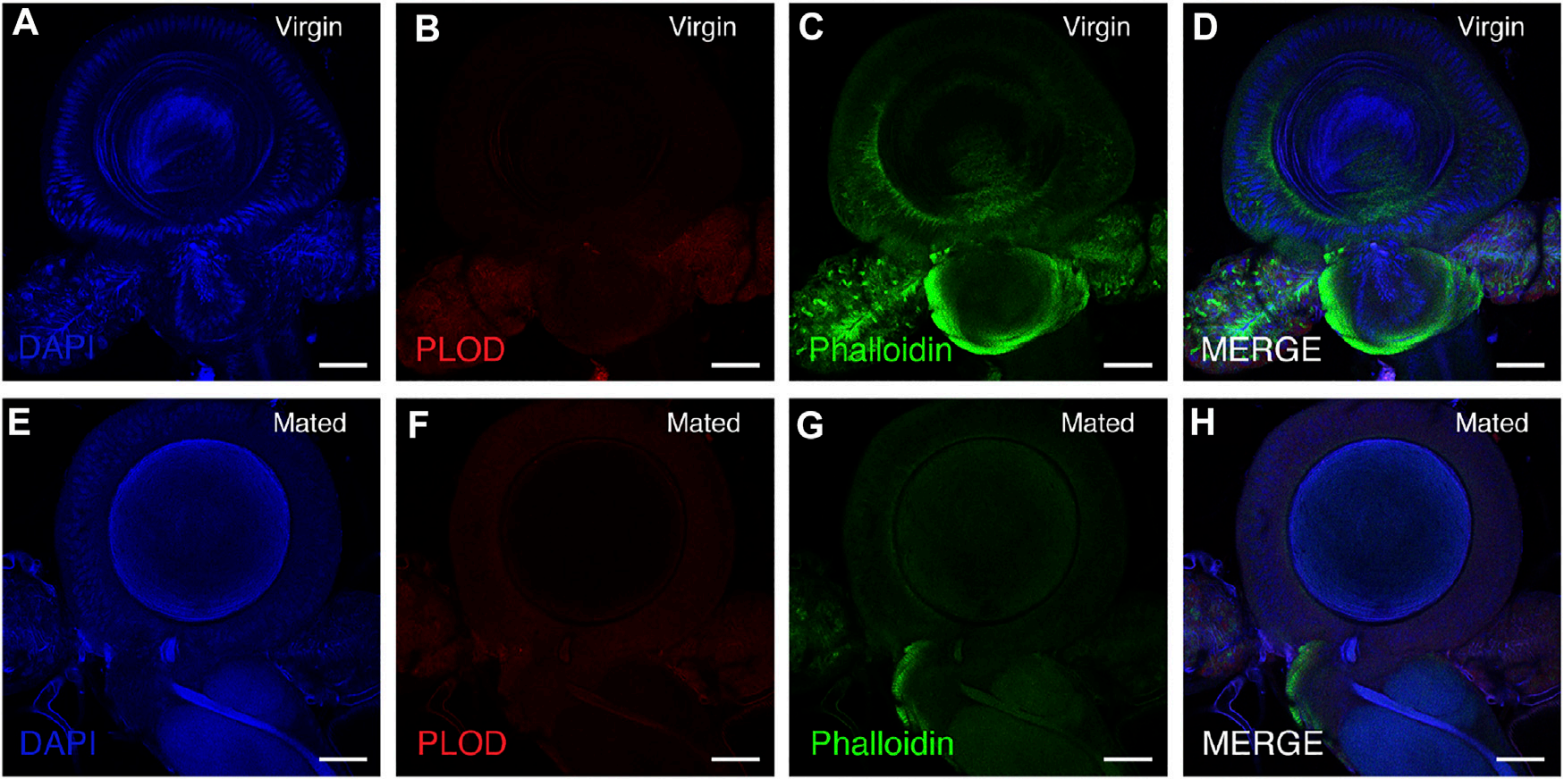
PLOD1 in spermathecae of virgin and mated queens of bumblebee, at 24 h post mating. PLOD1 was expressed at lower levels in the mated queen spermathecae **(F)** than in the virgin queen spermathecae **(B)**. In the panels, blue indicates DAPI staining, and green indicates Phalloidin staining. Scale bars, 75 µm.

**FIGURE 6:**
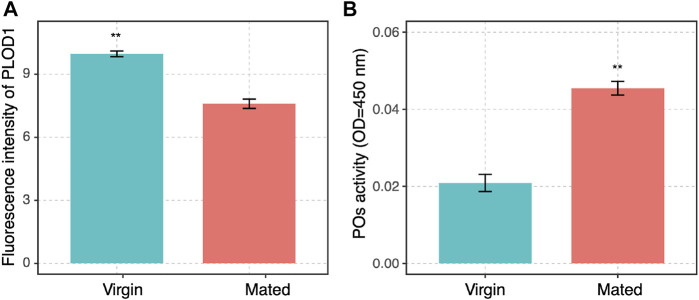
**(A)** The PLOD1 fluorescence intensity in spermathecae of mated and virgin queen was exhibited. The significant differences between the virgin (blue bars) and mated queens (red bars) are denoted with asterisks. **p < 0.01. **(B)** POs activity in spermathecae of virgin and mated queens of bumblebee, at 24 h post mating. The activity of PO was significantly increased in the spermathecae of mated bumblebee queens. The significant differences between the control group (virgin queens, blue bars) and mated queens (red bars) are denoted with asterisks. **p < 0.01. Three independent biological replicates were performed for each treatment.

A slight decrease in PLOD1 was observed in the mated queens spermathecae, as determined by RT-qPCR (Supplementary Figure S1). To further confirm the expression of PLOD1, we examined the PLOD1 levels in the spermathecae of virgin and mated bumblebee queens by IF, at 24 h post mating. Consistent with the RT-qPCR results, the level of PLOD1 in the mated queens spermathecae (Figure 5F) was dramatically decreased compared to that in the virgin (Figure 5B). The PLOD fluorescence intensity in spermathecae of mated and virgin queen was exhibited in Figure 6A.

### POs Activity of in the Spermathecae of Bumblebee Queens

The activity of PO in spermathecae of the mated queens significantly increased compared with the virgins (Figure 6B). POs are rate-limiting enzymes, and PO-mediated melanization plays a critical role in the insect immune system (Yassine et al., 2012; Binggeli et al., 2014; Ma et al., 2020). POs were also required for the *pea aphid*’s defense against bacterial and fungal infections (Xu et al., 2019). Therefore, the function of PO in spermathecae may protect sperm against infection.

## Discussion

In this study, RNA-sequencing was used to provide a global, high-throughput picture of the transcriptome of the spermathecae of bumblebee (*B. terrestris*) queens. The purpose was to start to address the molecular mechanism whereby bumblebee queens store viable sperm for several years after mating. This is the first report of a complete transcriptome of the spermathecae of mated and virgin bumblebee queens, in which over 10,000 genes were identified. The upregulated expression of immune response and sperm storage genes in the spermathecae of mated queens is likely to protect sperm during their long-term storage. The RT-qPCR data confirmed some of the genes expression differences at the mRNA level that were first identified in the RNA-sequencing data.

Most of the differentially expressed genes validated by RT-qPCR were concentrated on immune response (LRRC70, PO2, and Def) and sperm storage (TIL, Tret1, and hnRNP) function. Among these genes, those encoding leucine-rich repeat containing proteins (LRRC) are central to host innate defense in plants, invertebrates, and vertebrates (Kumar et al., 2014; Wu et al., 2019; Dominguez et al., 2020; Monti et al., 2020). In the haemolymph of bumblebee, *Bombus terrestris audax*, mating was found by significantly increases the abundance of antimicrobial peptides, including defensin, hymenoptaecin, and abaecin (Colgan et al., 2019). In insects, the biological activity of defensin is directed toward the protection against infectious diseases. The high expression of defensin in leaf-cutting ants, indicated that these ants invest in specific immune defenses for pathogen protection in organs that store sperm (Chérasse and Aron 2018). POs, which are rate-limiting enzymes, and considered as a core component in the insect immune system (Dudzic et al., 2015). In invertebrates, POs mediate important physiological processes, such as sclerotization, wound healing, and most important defense reactions (Noothuan et al., 2017; Rajendran and Vasudevan 2020; Unlu and Ekici 2021). POs are required in the *pea aphid* for survival against microbial infections (Yassine et al., 2012; Binggeli et al., 2014; Ma et al., 2020). Our RT-qPCR results showed that these immune response genes, including LRRC70, PO2, and Def play important roles in the spermathecae of bumblebee queen post mating (Figures 3, 4). The ELISA results showed that the activity of POs in spermathecae significantly higher than that in virgin queens (Figure 6B). The expressions of POs in the spermathecae of mated queens is likely to protect sperm during their long-term storage. Our findings demonstrated that the external sperm transfer into spermathecae led to the upregulative expression of immune response genes, but the detailed mechanisms need further experiments to validate.

The chymotrypsin family, which includes chymotrypsin inhibitors (TILs), is a large group of enzymes. Although these enzymes contain a highly conserved tertiary structural fold, they have developed a range of substrate specificities critical to many biological functions, including blood coagulation and immune responses (Dunse et al., 2010). TILs have been reported to decrease protein degradability, resulting in lower availability of amino acids and peptides for production purposes. *In vitro*, TILs were effective in preventing the acrosome reaction process (AR) induced by progesterone in canine spermatozoa (Deppe et al., 2010). The upregulated expression of TIL in mated bumblebee queens spermathecae may maintain protein stability. The Tret1 sequences were conserved in insects (Kanamori et al., 2010). The Tret1 is a highly specific transporter of trehalose, and trehalose may be a useful cryoprotective or dehydrating molecule for cells and biological molecules such as proteins and nucleotides. Tret1 has been previously reported to be related to the mating response (Huo et al., 2020). The hnRNP family was originally identified as nuclear RNA binding proteins, with the roles in processes such as mRNA processing and transport, transcription, DNA repair, and telomere maintenance mediated by DNA- or RNA-protein or protein-protein interactions, and many hnRNPs are involved in multiple cellular functions (Dreyfuss et al., 1993; Han et al., 2010; Ou et al., 2020). The transcription, splicing, stability, export through nuclear pores and translation of cellular and viral transcripts are all mechanisms modulated by hnRNP family protein (Jean-Philippe et al., 2013). The upregulated expression of hnRNP in mated bumblebee queens spermathecae may involve in sperm storage. Our RT-qPCR results demonstrated that these genes (TIL, Tret1, and hnRNP) may play major roles post mating (Figures 3, 4). The mRNA expression levels of TIL, Tret1, and hnRNP in the spermathecae of mated queens were dramatically increased at 24 h post mating, which suggested that these genes responded significantly to the sperm transfer. However, further experiments will be needed to elucidate the detailed mechanism.

PLOD1 catalyze the hydroxylation of lysine residues in collagens and other proteins containing collagenous-like domains. A slight decrease in PLOD1 was observed in the mated queen spermathecae, as determined by RT-qPCR, but the difference was not obvious. The detailed expression patterns of PLOD1 protein were also characterized by immunofluorescence (IF) in these organs, we observed that PLOD1 was lower expressed in the mated queen spermathecae than in the virgin one (Figures 5, 6A). The decreased expression of PLOD1 in the mated queen spermathecae might protect the sperm during the long-term storage and promote the mating behavior.

In conclusion, this is the first study to characterize gene expression in the spermathecae of bumblebee (*B. terrestris*) queens revealing the alterations in mRNA expression levels after mating. The high density of sperm in the spermatheca, as well as the low metabolic activity during storage, may have important functions in the sperm long-term storage. Further functional research on the expression of different genes should help to identify genes that are involved in long-term sperm storage in the queen spermathecae after mating and could potentially help to elucidate how these genes can affect bumblebee queen mating.

## Data Availability

The data presented in the study are deposited in the NCBI repository, accession number PRJNA779160.
