# Supplementary material for: Mating Stimulates the Immune Response and Sperm Storage-Related Genes Expression in Spermathecae of Bumblebee (Bombus terrestris) Queen
Source: Front Genet. 2021 Nov 26;12:795669. doi: 10.3389/fgene.2021.795669 (PMC8661091; doi:10.3389/fgene.2021.795669)
Supplement: Supplementary file 1 [file DataSheet2.docx]

**TABLE S1 |** RT-qPCR primers for these genes in this study, their sequences (5′ to 3′ sequence).

| **gene ID** | **LOC** | **gene name** | **Forward Primer** | **Reverse Primer** |
| --- | --- | --- | --- | --- |
| XM_003394269 | LOC100645555 | NRF | TTCACTTCGACGTGCGTAATACCT | ACAGCTCACAGCTCTCGCCA |
| XM_003394790 | LOC100642443 | LRRC70 | ATCGCCGGCAATCCCTGGTC | ACGTCGTCCTGTTCCGCACC |
| XM_020863096 | LOC110119301 | SPc | TGCCGGCTGGGGAAAGACAG | TCTGCCCGTAACCCAACGCC |
| XM_003395924 | LOC100631063 | Def | GGCAAAGCTGGAGGTCGCTG | ACGTCGAATAGTCGCGGCA |
| XM_003397252 | LOC100644683 | uncharacterized | ATTTGCGCGCTCTGTTCGGC | AGGTTTTGGCGTGGCAACCA |
| XM_020864904 | LOC100651168 | hnRNP-1 | CCCGTTATTGTTATACCGGTTGCAG | AGCTGGAACCTGCTTTCGCTG |
| XM_003398265 | LOC100651168 | hnRNP-2 | AGCGGAGGAGGCGGTGGATA | AGCTGGAACCTGCTTTCGCTG |
| XM_012314658.2 | LOC100645021 | WAP-1 | CGACCCACGACTGAGAGTCAACA | CGTACCTGGATTGAGCAAGCGT |
| XM_020865987 | LOC100645021 | WAP-2 | TCACGGCCAGGAATAACCAAGC | GACACGTTGTTCGACTTGCCGT |
| XM_003400500 | LOC100648607 | PO2 | ATCTGGTGCCCGAAGGTGCC | AGCGACGCGATGCTCGTCAT |
| XM_003401204 | LOC100645263 | TIL | AGCCCACTTGTGCCAAGCCA | TGCAAACGCCAGCGAGGAAG |
| XM_003401197.3 | LOC100644462 | Tret1-1 | CGTTGCTTGGGACTCGAAGCG | CGTCACGTCGAGCGAGAGTGT |
| XM_012317005.2 | LOC100644462 | Tret1-2 | GCCAGCTAAGGGCAAGAGAACC | GGGGATCGGCCAGTGGATTTACC |
| XM_003401198.3 | LOC100644462 | Tret1-3 | CGCCTGCGCTATATCGGCCT | CGGTCGGCTTGGTCGGGATT |
| XM_020866985 | LOC100644462 | Tret1-4 | TGAGGCACGGAAACGGTCCA | ACGTCCCGTCGCATCTGCTC |


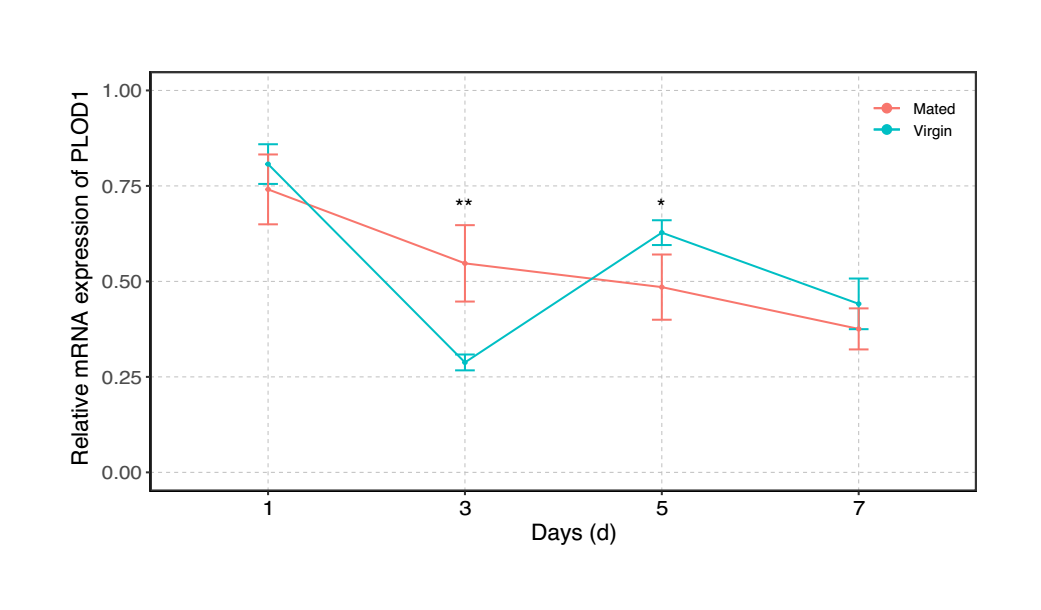


**FIGURE S1 |** Gene expression profiles of PLOD1 at different time points in mated and virgin bumblebee queens at 1-, 3-, 5-, and 7- day, as determined by RT-qPCR. The expression levels (Mean ± SEM) of PLOD1 in virgin queens (blue lines) and mated queens (red lines). Asterisks indicate significant differences between the expression profiles of mated and virgin queens. * p < 0.05; ** p < 0.01.
